# Improving Adherence to Antiretroviral Therapy for Youth Living with HIV/AIDS: A Pilot Study Using Personalized, Interactive, Daily Text Message Reminders

**DOI:** 10.2196/jmir.2015

**Published:** 2012-04-05

**Authors:** Nadia Dowshen, Lisa M Kuhns, Amy Johnson, Brian James Holoyda, Robert Garofalo

**Affiliations:** ^1^Craig-Dalsimer Division of Adolescent MedicineThe Children's Hospital of PhiladelphiaPhiladelphia, PAUnited States; ^2^School of MedicineUniversity of PennsylvaniaPhiladelphia, PAUnited States; ^3^Division of General Academic PediatricsChildren’s Memorial HospitalChicago, ILUnited States; ^4^Feinberg School of MedicineNorthwestern UniversityChicago, ILUnited States

**Keywords:** Adolescents, HIV/AIDS, adherence, text messaging, short message service, SMS, mobile health intervention

## Abstract

**Background:**

For youth living with human immunodeficiency virus (HIV)/acquired immunodeficiency syndrome (AIDS), nonadherence to antiretroviral therapy (ART) can lead to poor health outcomes and significantly decreased life expectancy.

**Objective:**

To evaluate the feasability, acceptability, and preliminary efficacy of short message service (SMS) or text message reminders to improve adherence to ART for youth living with HIV/AIDS.

**Methods:**

We conducted this prospective pilot study using a pre–post design from 2009 to 2010 at a community-based health center providing clinical services to youth living with HIV/AIDS. Eligibility criteria included HIV-positive serostatus, age 14–29 years, use of a personal cell phone, English-speaking, and being on ART with documented poor adherence. During the 24-week study period, participants received personalized daily SMS reminders and a follow-up message 1 hour later assessing whether they took the medication, and asking participants to respond via text message with the number 1 if they took the medication and 2 if they did not. Outcome measures were feasibility, acceptability, and adherence. Self-reported adherence was determined using the visual analog scale (VAS) and AIDS Clinical Trial Group (ACTG) questionnaire 4-day recall. Viral load and CD4 cell count were followed as biomarkers of adherence and disease progression at 0, 12, and 24 weeks.

**Results:**

Participants (N = 25) were mean age 23 (range 14–29) years, 92% (n = 23) male, 60% (n = 15) black, and 84% (n = 21) infected through unprotected sex. Mean VAS scores significantly increased at 12 and 24 weeks in comparison with baseline (week 0: 74.7, week 12: 93.3, *P* < .001; week 24: 93.1, *P* < .001). ACTG questionnaire 4-day recall also improved (week 0: 2.33, week 12: 3.24, *P* = .002; week 24: 3.19, *P* = .005). There was no significant difference in CD4 cell count or viral load between baseline and 12- or 24-week follow-up, although there was a trend toward improvement of these biomarkers and a small to moderate standardized effect size (range of Cohen *d*: –0.51 to 0.22). Of 25 participants, 21 (84%) were retained, and 20 of the 21 (95%) participants who completed the study found the intervention helpful to avoid missing doses.

**Conclusions:**

In this pilot study, personalized, interactive, daily SMS reminders were feasible and acceptable, and they significantly improved self-reported adherence. Larger controlled studies are needed to determine the impact of this intervention on ART adherence and other related health outcomes for youth living with HIV/AIDS.

## Introduction

Over one million people in the United States are living with human immunodeficiency virus (HIV)/acquired immunodeficiency syndrome (AIDS). Youth aged 12–29 years account for more than a third of the approximately 50,000 new HIV infections each year [1,2]. Nonadherence to antiretroviral therapy (ART) can lead to poor health outcomes and significantly decreased life expectancy, and it may increase the risk of secondary transmission and the development of resistant viral strains [3-7]. Challenges to adherence include pill burden, dosing schedule, food restrictions, and side effects [8-11]. However, forgetting is the most commonly cited reason for missing doses [12]. A range of strategies to improve adherence to ART have been shown to be helpful for youth living with HIV/AIDS, including directly observed therapy, reminder devices, counseling, and telephone calls, but many of these strategies are expensive, time consuming, and potentially intrusive [13-17].

With over 230 million cell phones in use and 7 billion text messages sent every month in the United States, text messaging or short message service (SMS) has become a common mode of communication among youth, including those who are economically disadvantaged [18]. This low-cost, convenient technology has provided benefit in a variety of health care settings and has been shown to be an effective tool for behavior change [19,20]. Evidence suggests that text messaging interventions may increase medication adherence among children and adolescents living with other chronic diseases such as asthma and diabetes [21-25]. Several studies have used both daily and weekly unidirectional, standardized SMS medication reminders for HIV-positive individuals in low-resource settings, but no published data have evaluated SMS medication reminders among youth living with HIV/AIDS in the United States [26-28].

In particular, text messaging is well suited as a vehicle for ecological momentary interventions; that is, mobile technology can provide treatment to patients in real time and in their natural environments [29]. Additionally, recent reviews of the literature on text messaging interventions for health behavior change have identified key characteristics for success, including interactivity and tailoring of messages, which were associated with higher retention rates in multiple studies [30].

The purpose of this pilot study was to evaluate the feasability, acceptability, and preliminary efficacy of an interactive text message reminder intervention for HIV-positive youth aged 14–29 years with demonstrated poor ART adherence. In particular, we sought to establish the feasibility of using personal cell phones for the intervention and our ability to retain youth living with HIV/AIDS as participants over the study period. We chose to pilot this intervention among youth with difficulty adhering to ART, since they could potentially benefit most from the reminders. We did not include a control condition given the lack of any pilot data to support this approach; therefore, the aims of this study were largely exploratory and meant to assess initial efficacy for a larger controlled trial.

## Methods

Eligibility criteria included HIV-positive serostatus, age 14–29 years, use of personal cell phone, English-speaking, and being on ART with poor adherence. We defined poor adherence as (1) missing more than 3 medication doses in the last month, or (2) missing any doses in the last month and not achieving viral supression after 24 weeks of an appropriate regimen, or (3) being referred from a clinician who documented poor adherence in the medical record defined by a report of any missed doses to any member of the care team or not achieving viral supression in the expected time period on an appropriate regimen. Possible participants were recruited primarily from a multidisciplinary program providing medical care and other support services to youth living with HIV/AIDS, located at a lesbian, gay, bisexual, and transgender-focused health center, and serving mainly young men who have sex with men of color who have acquired HIV through sexual activity. In this convenience sample, participants were recruited and enrolled consecutively from June to November 2009 and agreed to receive text messages over a 24-week study period. Participants received a US $40 incentive at each visit to compensate for time, transportation costs, and any additional charges incurred from the daily text messages.

At baseline, we collected demographic information and screened participants for depression and substance abuse, as these have been identified as factors affecting adherence [10,31]. We collaborated with Intelecare, a Health Insurance Portability and Accountability Act- and Health Information Technology for Economic and Clinical Health Act-compliant vendor of SMS health-related reminder services. At the time of enrollment, patients worked with the study coordinator to design their own personalized SMS reminder messages, which were programmed through Intelecare’s website to be delivered daily at the time(s) specified. Participants also designed a personalized follow-up message 1 hour later assessing whether they took the medication, and asking participants to respond via text message with the number 1 if they took the medication and 2 if they did not. Patients were encouraged to consider developing messages that would respect their privacy if they did not want to disclose their HIV diagnosis. Participants were able to contact the study coordinator to change the message at any time throughout the study period, and patients were asked to contact the study coordinator to reprogram the message if at any time their mobile service was interrupted.

**Figure 1:**
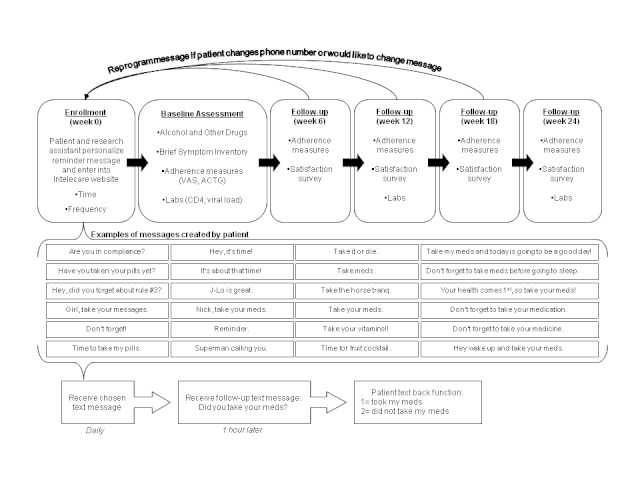
Study design. ACTG = AIDS Clinical Trials Group; Labs = laboratory tests; meds = medications; VAS = visual analog scale.

Follow-up visits at 6, 12, 18, and 24 weeks included assessments of adherence by visual analog scale (VAS) and the AIDS Clinical Trials Group (ACTG) adherence questionnaire and satisfaction surveys. The VAS prompts participants to rate adherence in the last 4 weeks on a scale of 0% to 100%. The VAS correlates highly with unannounced pill counts, 3-day adherence recall, and viral load (ie, *r* > .7) [32,33]. Additionally, participants responded to the following question from the ACTG adherence questionnaire (on a scale of 0 = never to 4 = all the time): “Over the past 4 days, how closely did you follow your specific medication schedule?” Viral load (HIV type 1 ribonucleic acid quantification) and CD4 cell count (absolute and percentage) were abstracted from the patient’s chart at baseline and at 12 and 24 weeks. See Figure 1 for details of the study design and Multimedia Appendix 1 for screenshots of the Intelecare platform used to send, recieve, and manage text message data. Study completion was defined as attendance at all study visits; responding daily to text messages was encouraged but not required for study completion.

Descriptive and distributional analyses were performed to describe the sample. Pre–post comparison of mean values and related effect sizes (standardized and unstandardized) were used to evaluate outcomes of interest. This pilot study was not powered to detect statistically significant differences. For assessment of pre–post changes in adherence levels and biomarkers, we compared scores using a *t* test for paired samples, with a significance level set at *P* ≤ .05 (2-tailed test), with Cohen *d* calculated as a standardized measure of effect size (controlling for dependency in paired values).

## Results

Participants were primarily male and of racial ethnic minorities, and had acquired HIV through unprotected sex (see Table 1). The main outcome of interest was change in adherence at the 12- and 24-week follow-ups (in comparison with baseline) as measured on the VAS. Mean VAS scores and responses on the ACTG 4-day adherence recall significantly increased at the 12- and 24-week follow-ups in comparison with baseline (see Table 2 and Table 3). On average, participants increased from a baseline value of 74.7 on the VAS to over 90.0 at both the 12- and 24-week follow-ups, with a standardized effects size (Cohen *d*) greater than 1.0 at both follow-up points. Improvements in adherence measures were seen as early as 6 weeks and sustained throughout the 24-week study period. There was no significant difference in CD4 cell count or viral load between baseline and the 12- or 24-week follow-ups, although there was a trend toward improvement of these biomarkers and a small to moderate standardized effect size (range of Cohen *d*: –0.51 to 0.22).

**Table 1:** Participants’ demographics characteristics (n = 25).

Characteristic		Mean or n	SD or %	Range
Age (years), mean (SD)		23	3.08	14–29
Time since diagnosis (months), mean (SD)		41	43.4^b^	7–180
Time since starting current ART^a^ regimen (months), mean (SD)		37	59.4^b^	1–240
**Gender, n (%)**				
	Male	23	92%	
	Female	2	8%	
**Race/ethnicity, n (%)**				
	Black	15	60%	
	White	2	8%	
	Latino	6	24%	
	Multiracial	2	8%	
**Mode of transmission, n (%)**				
	Perinatal	3	12%	
	Sexual contact	21	84%	
	Unsure	1	4%	
**Medication regimen frequency, n (%)**				
	Daily	20	80%	
	Twice daily	5	20%	

^a^ Antiretroviral therapy.

^b^ Time on ART longer than time since diagnosis reflects delayed disclosure of diagnosis to perinatally infected youth.

**Table 2:** Baseline and follow-up adherence and biomarker outcomes (n = 21 of 25).

Outcome measure	Baseline		12 weeks			24 weeks		
	Mean	SD	Mean	SD	*P* value	Mean	SD	*P* value
Adherence (VAS^a^)	74.7	16.5	93.3	6.6	<.001	93.1	7.7	<.001
Prior 4-day adherence (ACTG^b^)	2.33	1.1	3.24	0.4	.002	3.19	0.9	.005
Viral load	2750.2	8930.8	240.5	521.1	.26	28.5	47.5	.23
CD4 cell count	501.5	239.2	552.8	234.3	.12	544.8	228.7	.37

^a^ Visual analog scale.

^b^ AIDS Clinical Trials Group. Response scale: 0 = never, 4 = all the time.

**Table 3:** Effect sizes and changes in adherence and biomarker outcomes from baseline to 12 and 24 weeks (n = 21 of 25).

Outcome measure	12 weeks				24 weeks			
	Change	95% CI^a^	*P* value	Effect size (Cohen *d*)	Change	95% CI	*P* value	Effect size (Cohen *d*)
Adherence (VAS^b^)	18.6	10.4–27.0	<.001	1.18	18.5	10.5–26.5	<.001	1.13
Prior 4-day adherence (ACTG^c^)	0.9	0.4–1.4	.002	0.87	0.9	0.3–1.4	.005	0.73
Viral load^d^	–2509.7	–7067.0 to 2047.6	.26	–0.41	–2721.7	–7306.7 to 1863.3	.23	–0.51
CD4 cell count^d^	51.3	–15.05 to 117.6	.12	0.4	43.3	–56.3 to 142.9	.37	0.22

^a^ Confidence interval.

^b^ Visual analog scale.

^c^ AIDS Clinical Trials Group. Response scale: 0 = never, 4 = all the time.

^d^ Sample size n = 17 for viral load and CD4 cell count due to additional missing participants at 24 weeks.

Enrollment and retention of the study population was feasible with 25 participants enrolled over a 6-month period (approximately 4 participants enrolled per month), and with 23 of 25 (92%) and 21 of 25 (84%) of participants completing all visits at 12 and 24 weeks, respectively (see Table 4). Of the 4 participants who did not complete the study, only 3 were lost to follow-up; 1 had to be removed because of cell phone service incompatibility with Intelecare technology. Of note, several participants experienced a lapse in cell phone service (some due to change in phone number, failure of payment, or a lost phone, and 1 patient had the mobile phone stolen during an assault), and none reported it directly to the study coordinator, but all reported the interruption to their medical provider or case manager during routine care, and the study coordinator was then notified and messages were reprogrammed.

The intervention was rated highly on indicators of satisfaction: at the 24-week follow-up, 17 of 21 (81%) participants who completed the study said they would like to continue to receive text messages after the end of the study, and 20 of 21 (95%) participants indicated that the text messages helped them “very much” to miss fewer doses of medication. Reasons why participants did not find reminders helpful included being at work during the day when they received the text message and wanting to take their medications but not being able to check messages; not having medications on hand; living temporarily at a friend’s house where they were unable to store medications; and being in a public place where they felt uncomfortable taking their medications. Of note, all participants who completed the study (n = 21) felt that the intervention would have been helpful when they first started taking medication.

During the study period 15,387 messages were sent and received through the Intelecare platform. Of the outgoing messages sent 1167 messages were not delivered and 14,220 messages were successfully sent. Of the 7110 messages requesting a response, 3414 (48.02%) text message replies were sent by participants indicating whether they took their medications.

**Table 4:** Feasibility and acceptability of the study intervention.

		n	%
**Feasibility (n = 25)**			
	Rate of study completion	21	84%
**Acceptability (n = 21)**			
	Helpful to avoid missed doses?	20	95%
	Helpful to remember refills?	16	76%
	Helpful to remember medical appointments?	15	71%
	Messages respected privacy?	21	100%
	Received all messages?	17	81%
	Would like to continue to receive reminders?	17	81%
	Reminders would have been helpful when starting medications?	21	100%

## Discussion

In this sample of 25 HIV-positive youth with previous difficulty adhering to ART, personalized, daily text messaging significantly improved self-reported adherence from baseline to 12 and 24 weeks. Retention in the study was excellent, participants reported high levels of satisfaction, and the vast majority wanted to continue receiving reminders after the 24-week study period was completed. The lack of a significant difference in biomarkers (CD4 cell count and viral load) was likely due to the small sample size.

Major limitations of the study include absence of a control group and lack of long-term follow-up after completion of the intervention. However, given the paucity of data showing any effective intervention to improve adherence among youth living with HIV/AIDS, the pre–post difference reported in this study is an important finding. In one study that did show improvement with weekly text reminders and no improvement with daily reminders among poorly adherent adults living with HIV/AIDS, patients in the control group had worse virologic and self-reported adherence than at baseline [26]. In the present study, rates of self-reported adherence and virologic suppression improved beginning at 6 weeks and were sustained over the 24-week study period, in contrast to Pop-Eleches and colleagues’ study, where standardized weekly and daily texts had a waning effect on self-reported adherence and treatment interruptions over time [26]. A recent randomized controlled trial of 19 adults living with HIV with poor adherence showed a similar improvement in adherence by self-report for those participants randomly assigned to text message reminders personalized by topic versus no change in adherence for those randomly assigned to a reminder beep on a pager [34]. This study followed patients for 6 weeks and did not include any biomarkers for adherence. The similar rates of improved self-reported adherence sustained over a longer period in our study, along with a trend toward improvement and a moderate standardized effect size for the difference in viral load from baseline to 24 weeks, further support the possible utility of this intervention.

Additionally, previous studies of text messaging interventions to improve adherence have used an interactive function and tailored messages, but none have included a daily, real-time interactive feature, and messages were not created individually by participants. This study showed that it is feasible to employ this intervention for a difficult-to-reach population using their own mobile phones. The unique, interactive feature of this intervention may provide additional information about timing of missed doses and help to uncover barriers to adherence, other than simply forgetting, that medical providers could use to problem solve with patients. For example, algorithms could be programmed so that if a patient did not respond for a certain number of days the provider and patient could be alerted to contact each other and address any issues in real time as opposed to waiting for the next routine clinic visit.

 In summary, this pilot study demonstrates that a daily, interactive, personalized text message reminder intervention is both feasable and acceptable and shows promise as a tool to help HIV-positive youth adhere to ART. Larger controlled studies are needed to determine the potential of this intervention, not only to improve adherence to ART, but also to affect a broad range of related health outcomes for youth living with HIV/AIDS.

## Multimedia Appendix 1

Intelecare platform for creating, sending, receiving, and managing text messages.

## Abbreviations

ACTG: AIDS Clinical Trial Group
AIDS: acquired immunodeficiency syndrome
ART: antiretroviral therapy
HIV: human immunodeficiency virus
SMS: short message service
VAS: visual analog scale
