# Supplementary material for: Improving Adherence to Antiretroviral Therapy for Youth Living with HIV/AIDS: A Pilot Study Using Personalized, Interactive, Daily Text Message Reminders
Source: J Med Internet Res. 2012 Apr 5;14(2):e51. doi: 10.2196/jmir.2015 (PMC3376506; doi:10.2196/jmir.2015)
Supplement: Supplementary file 1 [file jmir_v14i2e51_app1.pdf]

Intelecare®
from remedy health media

Adherence**Intervention**

Improving Adherence to Antiretroviral Therapy for Youth Living with HIV/AIDS (YLH): A pilot study using personalized, interactive, daily text message reminders

Intelecare Overview

- Modular, private label patient reminder suite scales to meet client adherence needs (online & mobile)

- Extend functionality with educational content, step programs, rewards, call center console and mail order pharmacy

- Leverage millions of patients interested in receiving information about health conditions, brands and offers for lead gen

- Client roster includes managed care, pharma, pharmacies, hospitals, academic medical centers

- 12.6mm covered lives and average over 5mm messages delivered daily

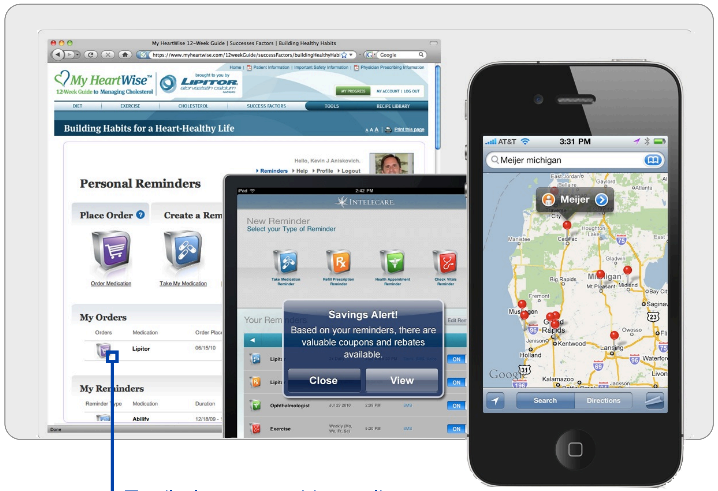

Easily Integrated into client websites and mobile apps

Admin Management Console

Custom built console

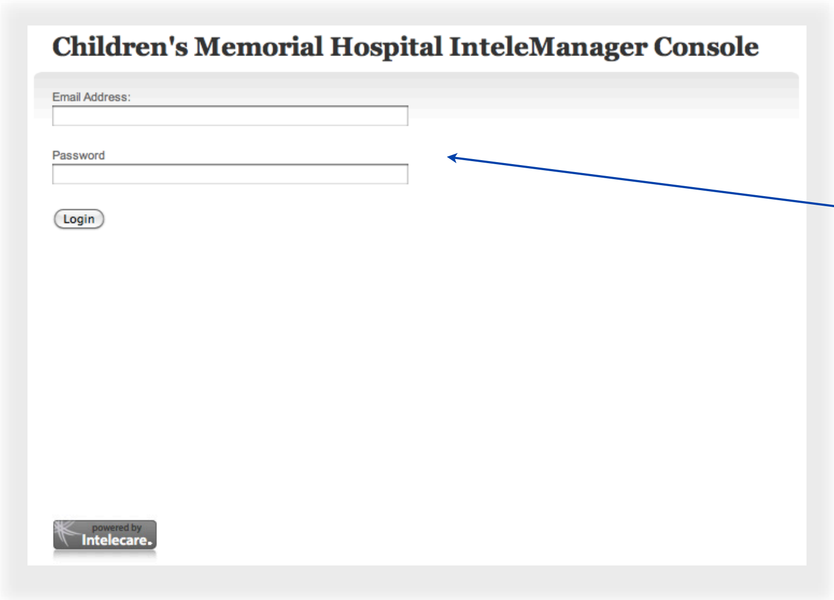

Access only granted to study coordinators or administrators

Manager Console

Login allows the admin access to the Manager Console

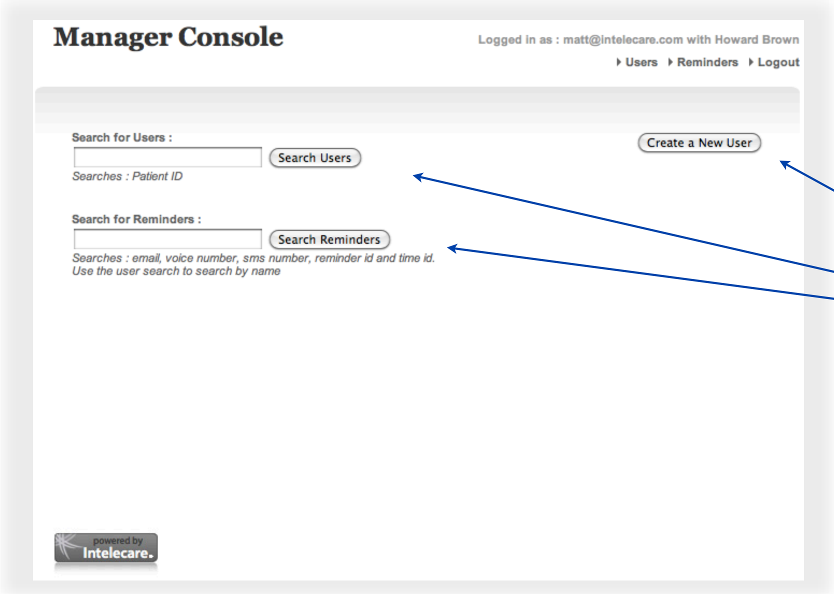

Admins can Search Users, Search Specific Reminders, Create and Manage Users

User Management: Participant List

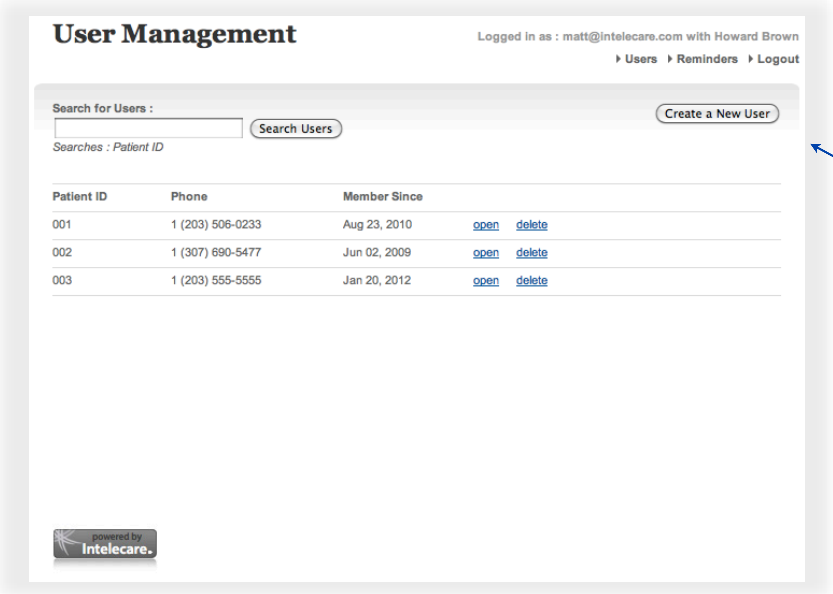

Searching users pulls up a list of participants

Selecting "Create a New User" allows you to enter a new subject into the system

User Management: Creating a New User

Simple, quick process for Creating a New User

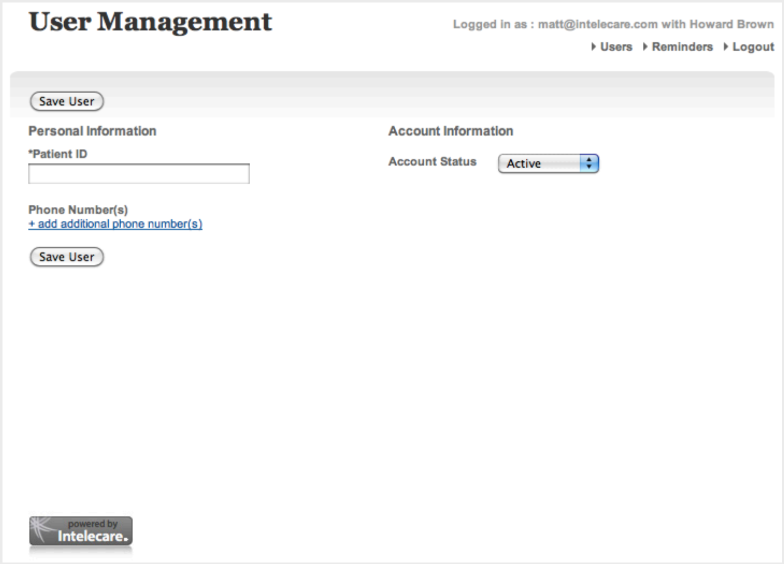

User Management: Creating a New User

ID numbers used to protect the users' identity

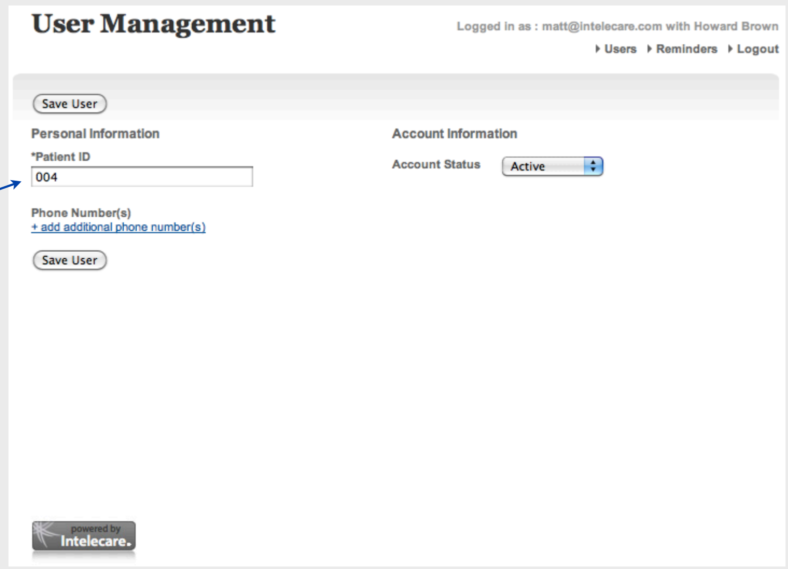

User Management: Creating a New User

Mobile number uploaded

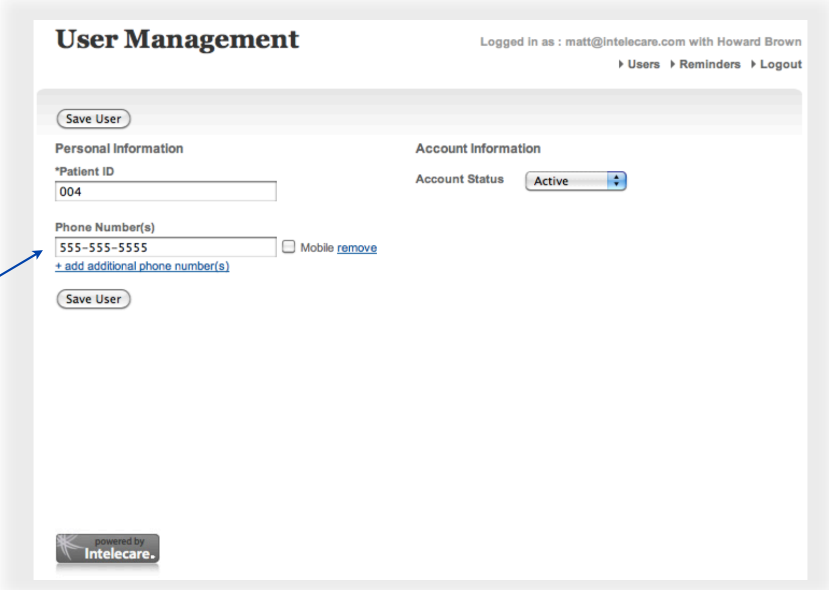

Intelecare, from Remedy Health Media
Proprietary & Confidential // Not for External Use or Distribution

User Management: Creating a New User

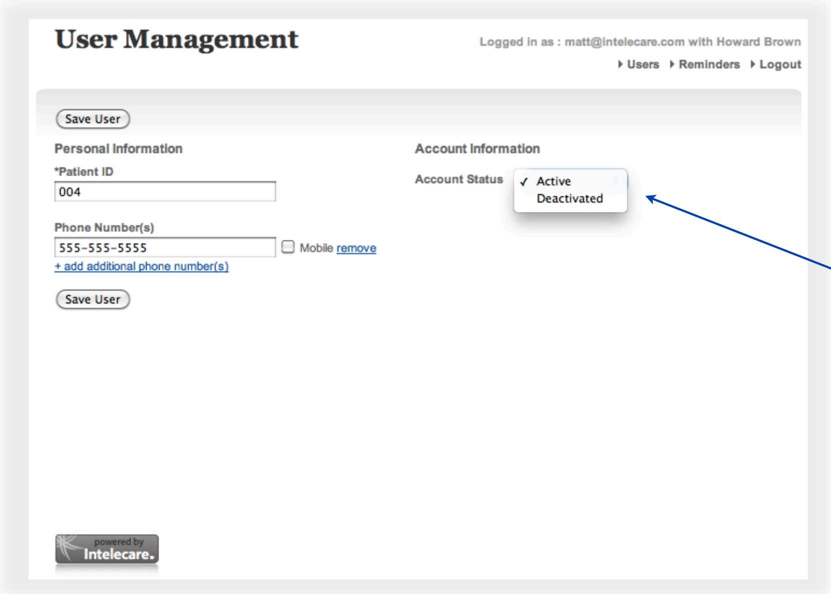

Users can be activated or deactivated depending on status in the study

User Management: User Account

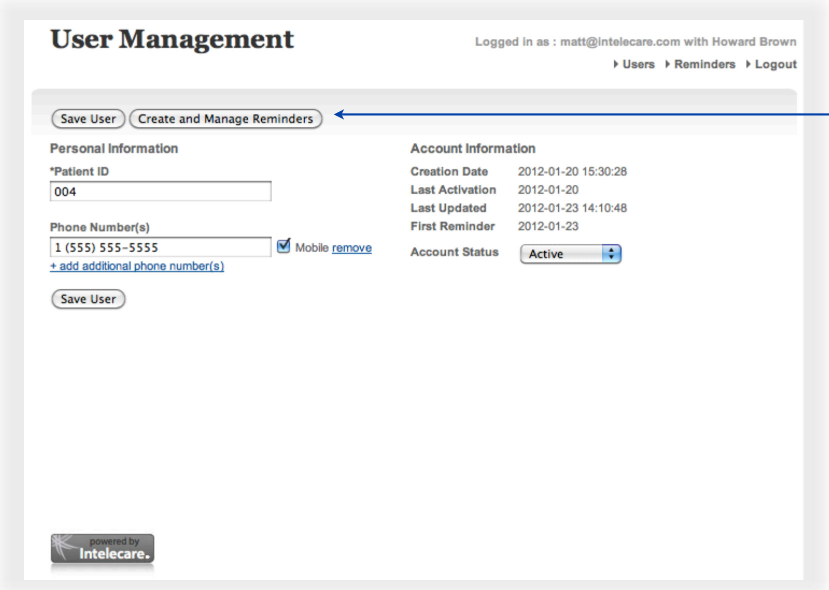

Once a new user is created and saved, reminders can be managed from this page

User Management: User List

Once a new User is created and saved, they become accessible in the user/subject list.

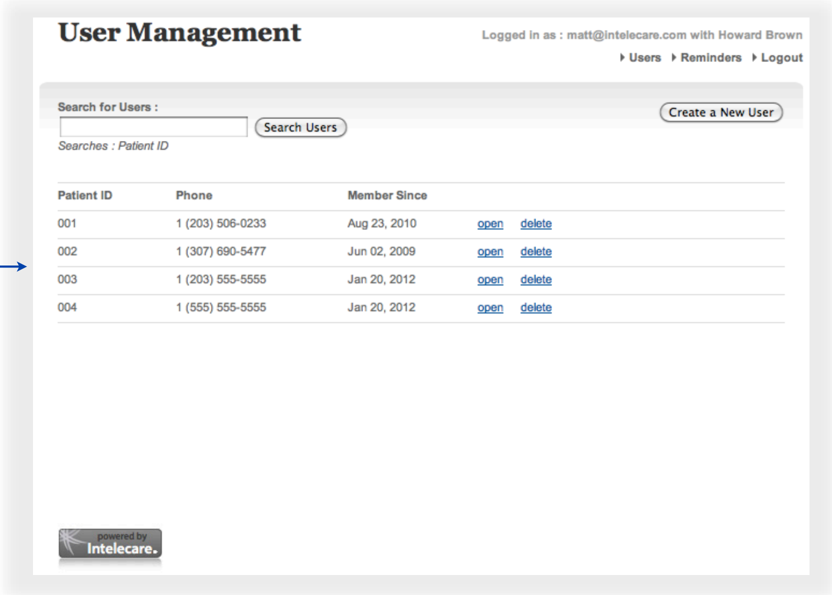

All user data is encrypted and securely stored on Intelecare servers.

Reminder Management: Creating Reminders

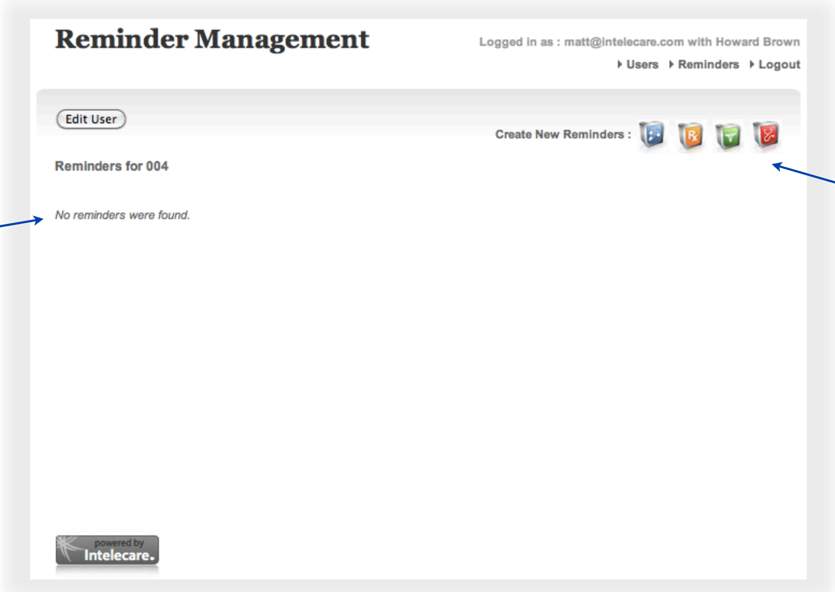

New user showing no reminders. As new reminders are created, a list will populate here.

Reminders can be created to Take or Refill your medications, for Doctor's appointments or other Lifestyle needs.

SMS Reminders: Scheduling Interface

Simple, intuitive interface to schedule reminders

All reminder "Types" follow the same creation process for ease of use

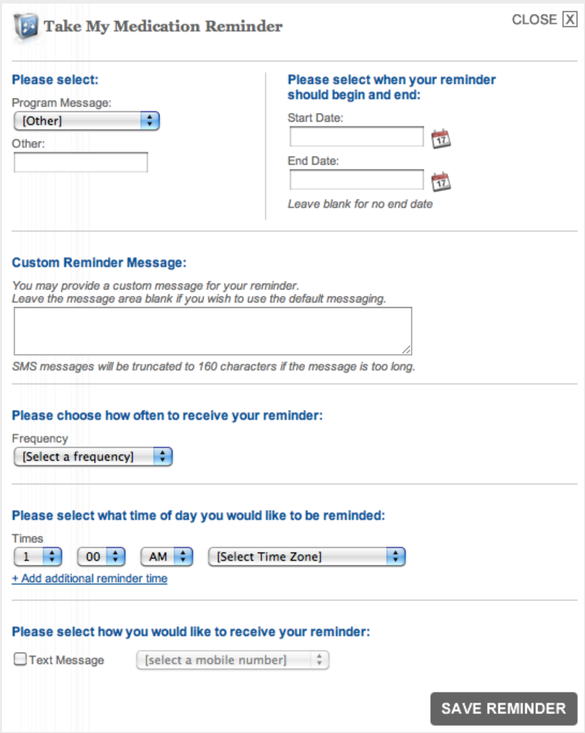

SMS Reminders: Custom Reminder Message

Reminder message is custom written per the subject's or admin's preference.

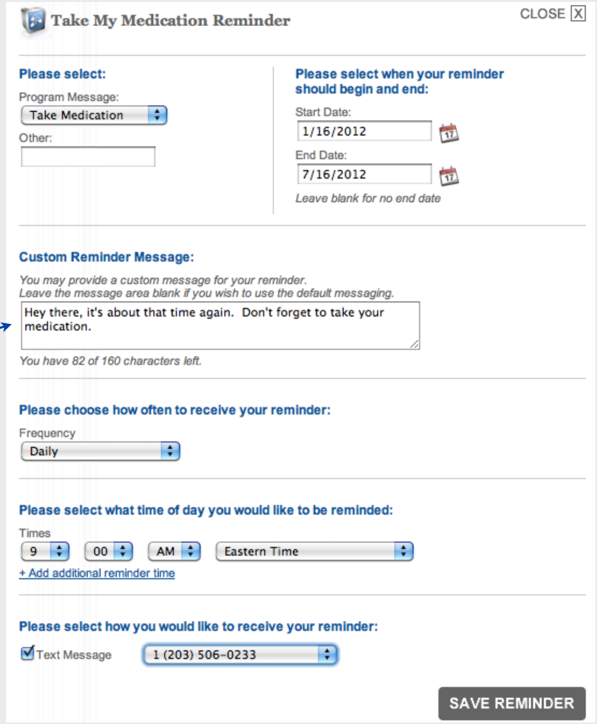

SMS Reminders: Reminders List

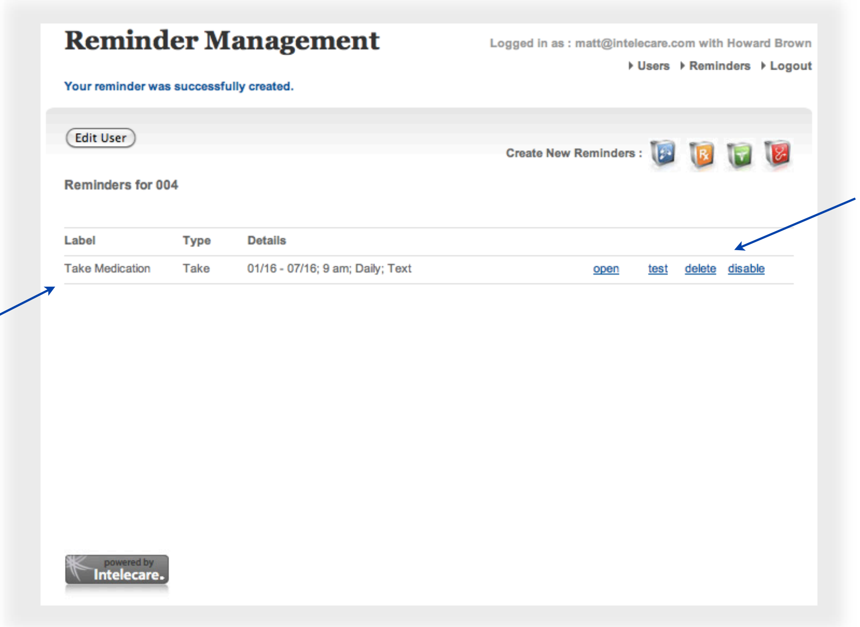

Once saved, reminder is populated to the individual's Reminder List

Saved reminders can be edited, disabled or deleted. Delivery can also be tested immediately after completion to confirm the user will receive the messages.

SMS Reminders: Confirmation Message

This study also included a follow-up, or Confirmation Message.

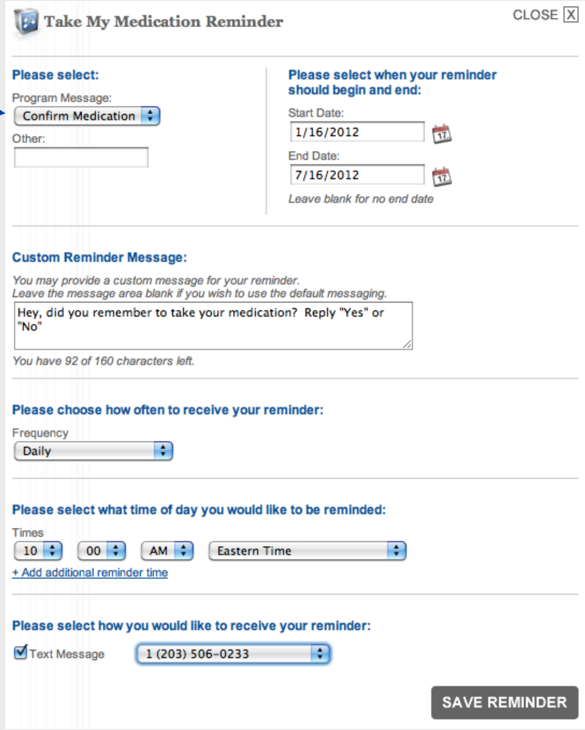

SMS Reminders: Reminder List

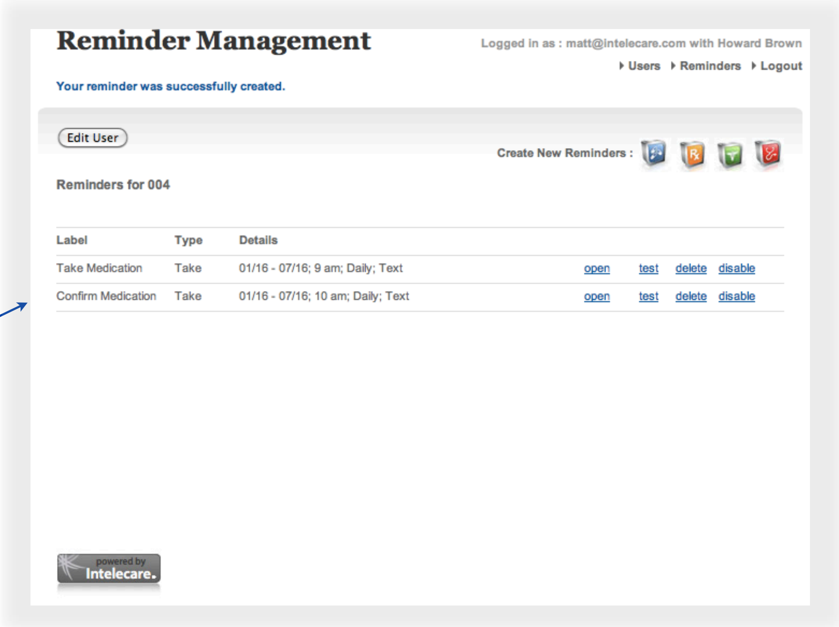

Again, completed reminders are saved to the Reminder List.

Reminder Delivery: Take

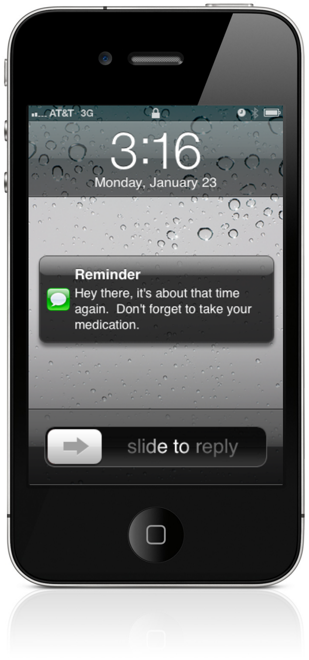

SMS messages are delivered to the user's device. Intelecare SMS can be accepted by virtually all Mobile Carriers.

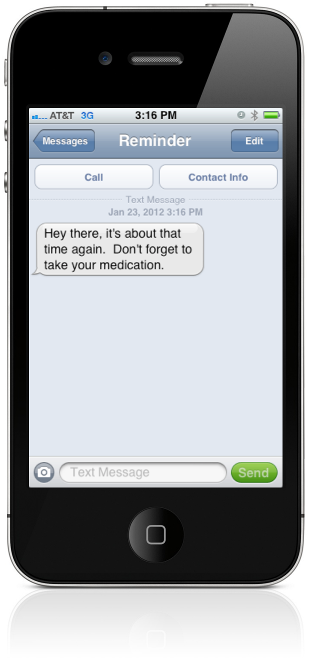

Reminder Delivery: Confirmation

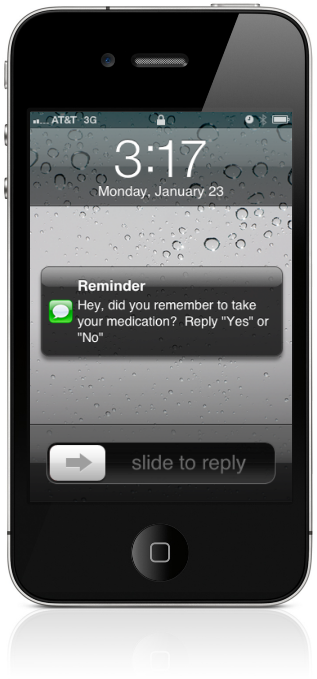

Confirmation message sent after initial reminder. Prompts user to reply for compliance tracking.

All responses are collected and stored.

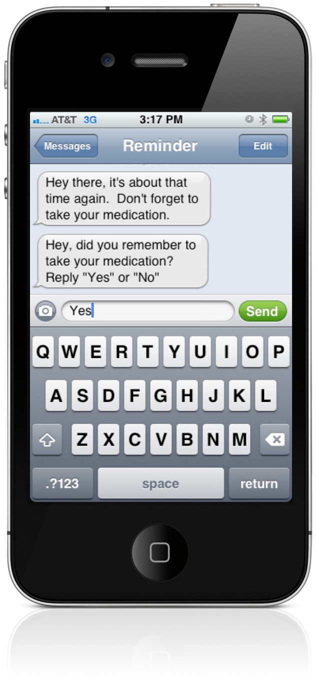

Data Recovery

Sample Data
Form received from
SMS system

Highlighted lines show users' response to the confirmation message

Phone Number	Delivery	Time	Message	Status
312-xxx-xxxx	Outgoing	9/11/09 1:00	don't forget to take your medication	sent
312-xxx-xxxx	Outgoing	9/11/09 2:00	did you take it, respond "1" for "yes", respond "2" for "no"	sent
312-xxx-xxxx	Incoming	9/11/09 2:01	1	Received
312-xxx-xxxx	Outgoing	9/12/09 1:00	don't forget to take your medication	sent
312-xxx-xxxx	Outgoing	9/12/09 2:00	did you take it, respond "1" for "yes", respond "2" for "no"	sent
312-xxx-xxxx	Incoming	9/12/09 2:05	1	Received
312-xxx-xxxx	Outgoing	9/13/09 0:59	don't forget to take your medication	sent
312-xxx-xxxx	Outgoing	9/13/09 2:00	did you take it, respond "1" for "yes", respond "2" for "no"	sent
312-xxx-xxxx	Incoming	9/13/09 2:02	1	Received
312-xxx-xxxx	Outgoing	9/14/09 0:59	don't forget to take your medication	sent
312-xxx-xxxx	Outgoing	9/14/09 1:59	did you take it, respond "1" for "yes", respond "2" for "no"	sent
312-xxx-xxxx	Incoming	9/14/09 2:007	1	Received
312-xxx-xxxx	Outgoing	9/15/09 0:59	don't forget to take your medication	sent
312-xxx-xxxx	Outgoing	9/15/09 2:00	did you take it, respond "1" for "yes", respond "2" for "no"	sent
312-xxx-xxxx	Incoming	9/15/09 2:02	2, not yet	Received
312-xxx-xxxx	Outgoing	9/16/09 1:00	don't forget to take your medication	sent
312-xxx-xxxx	Outgoing	9/16/09 2:00	did you take it, respond "1" for "yes", respond "2" for "no"	sent
312-xxx-xxxx	Incoming	9/16/09 2:02	1	Received
312-xxx-xxxx	Outgoing	9/17/09 1:00	don't forget to take your medication	sent
312-xxx-xxxx	Outgoing	9/17/09 2:00	did you take it, respond "1" for "yes", respond "2" for "no"	sent
312-xxx-xxxx	Incoming	9/17/09 2:15	1, on it	Received
312-xxx-xxxx	Outgoing	9/18/09 1:00	don't forget to take your medication	sent
312-xxx-xxxx	Outgoing	9/18/09 1:59	did you take it, respond "1" for "yes", respond "2" for "no"	sent
312-xxx-xxxx	Incoming	9/18/09 2:01	1	Received

Contact Details

Intelecare, from Remedy Health Media
500 5th Avenue, 19th Floor | New York, New York 10110

Matthew Pepe

Main (212) 994-9395
Telefax (212) 695-2936
Email mpepe@remedyhealthmedia.com

RemedyHealthMedia.com
